# Supplementary material for: A Qualitative Evaluation of a Health Access Card for Refugees and Asylum Seekers in a City in Northern England
Source: Int J Environ Res Public Health. 2023 Jan 12;20(2):1429. doi: 10.3390/ijerph20021429 (PMC9859311; doi:10.3390/ijerph20021429)
Supplement: Supplementary file 1 [file ijerph-20-01429-s001.zip › ijerph-2028879-supplementary.pdf]

International Journal of
Environmental Research and Public Health

A Health Access Card for refugees and asylum seekers in Newcastle upon Tyne: A qualitative evaluation

Interview schedule ***(semi-structured interviews with service users)***

1. Thank the participant for the interest they have shown in the evaluation. Describe the purpose of the evaluation – to explore the impact and usefulness of a health access card launched last February, and to discuss in more general terms the participant's experience of accessing health services in Newcastle – and ensure that the participant has read and understood the participant information sheet. Introduce oneself and describe background.

   Remind the participant that their involvement in the research is confidential, that they will be anonymised in any written/published literature that emerges from the evaluation, referred to only by a pseudonym, and that they can withdraw at any point, including during the interview. However, any materials obtained up to the point of withdrawal will be retained. Explain that the interviewer will be audio recording the interview to have an accurate record of what the participant has said, but that said recording will be destroyed following completion of the evaluation.

   Ask the participant if they have any questions about the study and remind them that they will have the opportunity to ask questions at the end of the session about anything they have not understood. Ask the interpreter if they have any questions (if applicable).

   If the participant is happy to proceed with the interview, obtain signed consent and begin the interview.

2. **Familiarity with the card**

   Do you have/were you a given a health access card?

   If **YES**, can you describe how you have used the card?

   If **NO**, the interviewee will be shown a copy of the card and asked to describe their initial impressions.

3. **Usability**

   - How easy is it to read and understand the information on the card?
   - What do you like about the card?
   - What information on the card is new to you, or particularly helpful?
   - To what extent have you shared the information on the card with your family or wider social network?

- Have friends or relatives benefitted from using the card or have they commented on the usefulness of the card?

4. **Areas for improvement**

- How might we improve the card?
- What other information about health services in Newcastle might be useful to include on the card?

5. **Experience of accessing health services**

- What health services have you used since arriving in Newcastle?
- How would you describe your experience of using these services?
- Did you face any barriers to accessing these services, and if so, can you describe them? Have there been times that you feel that you have not been able to access the services you need?
- In what ways have you been supported to access appropriate health services since you arrived in Newcastle?
- Do you feel that all of your health needs are currently being met?
- Have friends or family reported any particularly positive or negative experiences of health services in Newcastle, and if so, can you describe them?

6. **Improving access to health services**

- What additional support would you like to be better able to access health services in Newcastle?
- How might services in Newcastle be adapted/improved to be more responsive to your needs?
- Do you have anything else you would like to say about the health access card or about health services in the city that might be useful to our evaluation?

Inform the participant that this concludes the interview. Thank them for their participation and offer to answer any questions they have.

A Health Access Card for refugees and asylum seekers in Newcastle upon Tyne: a qualitative evaluation

Topic guide ***(focus group session with professionals)***

7. Thank the participants for the interest they have shown in the evaluation. Describe the purpose of the evaluation – to explore the impact and usefulness of the health access card launched last February, and to discuss in more general terms the participants' experience of how refugees and asylum seekers

access health services in Newcastle – and ensure that the participants have read and understood the participant information sheet. Introduce oneself and describe background.

Remind the participants that their involvement in the research is confidential, that they will be anonymised in any written/published literature that emerges from the evaluation, referred to only by a pseudonym, and that they can withdraw at any point, including during the focus group session. However, any materials obtained up to the point of withdrawal will be retained. Explain that the interviewer will be audio recording the session to have an accurate record of what the participants have said, but that said recording will be destroyed following completion of the evaluation. Participants will be asked not to share the details of the discussion and not to repeat the comments of other participants outside of the session.

Ask the participants if they have any questions about the evaluation and remind them that they will have the opportunity to ask questions at the end of the session about anything they have not understood. Inform them that one researcher will facilitate the discussion and that a second researcher will be present to take notes and to deal with any unforeseen problems that may arise.

If the participants are happy to proceed with the focus group, obtain signed consent and begin the session.

8. Familiarity with the card

Are all of the participants familiar with the card?

If **YES**, ask the participants to briefly describe their involvement in the development, launch and distribution of the card.

If **NO**, all participants will be shown a copy of the card and participants who answered YES will be asked to briefly describe their involvement in the development, launch and distribution of the card.

9. Usability and impact

- What are your thoughts about the usefulness and impact of the card?
- What has been the response among service users to the card?
- What information on the card has been new to service users, or particularly helpful?
- To what extent has the card been effectively distributed?
- In what ways have service users changed their approach to accessing health services since the launch of the card?
- How might the card be improved? What additional information might be included on future versions of the card?

10. Service users' experience of accessing health services

- To what extent do you feel that service users' health needs are currently being met?
- What are the particular challenges and barriers that service users face in accessing health services in Newcastle?
- What is the mental health experience of service users in Newcastle?
- How are service users currently supported to access health services?
- Have service users reported any particularly positive or negative experiences of health services in Newcastle, and if so, can you briefly describe them?
- Has COVID-19 presented any unique challenges to refugees and asylum seekers in terms of how they access health care?

11. Improving access to health services

- What additional support would help refugees and asylum seekers to be better able to access health services in Newcastle?
- How might services in Newcastle be adapted/improved to be more responsive to service users' needs?
- Do you have anything else you would like to say about the health access card or about health services in the city that might be useful to our evaluation?

Inform the participants that this concludes the focus group session. Thank them for their participation and offer to answer any questions they have.
